# Supplementary material for: Patterns of Intron Gain and Loss in Fungi
Source: PLoS Biol. 2004 Nov 30;2(12):e422. doi: 10.1371/journal.pbio.0020422 (PMC532390; doi:10.1371/journal.pbio.0020422)
Supplement: Table S1 — Also available at http://genes.mit.edu/NielsenEtAl/. (4.3 MB ZIP). [file pbio.0020422.st001.zip › NielsenEtAl/html/1338.html]

AN5763.1.NCU07960.1.MG05085.1.FG00405.1


```
 CLUSTAL W (1.82) Multiple Sequence Alignments - Introns Inserted


Sequence 1: MG05085.1	593 aa
Sequence 2: FG00405.1	572 aa
Sequence 3: NCU07960.1	595 aa
Sequence 4: AN5763.1	500 aa
Alignment Length: 622 aa
Number Identitical Residues: 166 aa
Alignment Score (without introns) 10776


MG05085.1 	MGLGILEDRHMDHVPG1----TTRHYDDPNRPTRVGEG-ATTLKTT---EDGIIL~VPQP
NCU07960.1	MGLGILEDRVLEHVPA1NLSGTTQYFDDPERPQYATDGAHEHLKCDRSGPVPIVL~VPQP
FG00405.1 	MPLGILEAKDIEHVPG1----TTRYFDDPSQPQTADEQ-HGNLKRVRVGEETIIL0IPQP
AN5763.1  	MGLGIVDVKGRQ-VPG1----TVDIYEK-ERVESQPSSYHTELKYDRSGSEPILL~VPQP
          	* ***:: :  : **.     *.  ::. .:     .     **    .   *:* :***

MG05085.1 	SDDPNDPL~NWPLWKRDLITAILSMTAI2FATSLGPILAANTITLS-LWFGQNLTKIAIL
NCU07960.1	TDDPNDPL~NWPLWRRDLITLVLSITSV~FATALGPILAADTVLLVSTLKDVTFVKASLL
FG00405.1 	SDDPNDPL~NWSLLRRDLITFLLCFAGI~LATALGPILAANTITIS-LLFAKDFTKVALL
AN5763.1  	SDDPNDPL0NWPLWKRDLTIFALSLVAV~LCATTSSVMAAGTVTLS-RYYRKSFMSIALL
          	:******* **.* :***    *.:..: :.:: ..::**.*: :        : . ::*

MG05085.1 	TGYFLLGVGAAGTFFVPSSRIWGKRHAFVVGLLILIGSSAWGGAVY-KNYDSMVGARIIQ
NCU07960.1	TGYFLLGCGASAFLFVPSSRIWGKRHLFVLGTILLIVTSIWTAASGNKSYTNFLWSRIFQ
FG00405.1 	TGYFLLGCGAGAIFFVPSGRIWGKRHLFLIGILIIIASSAWAGAVG-TNYGSFIGARIVQ
AN5763.1  	TGYHLCGTGIAGILIVPTARVWGKRHLFVLGHTLMVVSCVWAGASG-LNYQSLLWARIFQ
          	***.* * * .. ::**:.*:***** *::*  ::: :. * .*    .* .:: :**.*

MG05085.1 	GVGAAPYEALVNAAVGDLYFVHQRGKRMAFTNLAVFGGAFFTPILVG1VITHSIMW0WWT
NCU07960.1	GVAAAPFESLVNAAVGDLYFVHERGKRMAFTNLAVFGGAFFTPILVG~KISNDMGW~QWT
FG00405.1 	GVGCAPYESFLNAAVGDLYFVHQRGVRMAFTNLAVFGGAFFTPILVG~KITHEMGW~KWT
AN5763.1  	GVALAPFEALTNAVVDDLFYVHQRGKRMALSNVAVFGAAFLTPVAA-~--------~---
          	**. **:*:: **.*.**::**:** ***::*:****.**:**: .              

MG05085.1 	FYIVAILCA~ACLPLVVFFCPETAYKRDQS-------------LNTD----MGVDFQTGH
NCU07960.1	FWFVAIFSG~VCLPAVYLFCPETAYRRDPTPRFSATKKDDDDAPQKDRGLDQEQDQELKQ
FG00405.1 	FYFVAIFLA~ATLPAVFFLCPETAYRREAS-------------LNTDTTGELGIELTTKD
AN5763.1  	--------A1AALPLTIFLVPETAFRRPNY-------------LELD---FQDRDRTESR
          	        . . ** . :: ****::*                 : *       :     

MG05085.1 	QLRTFSEGSDSPPAQGQSTADEEK-LVGRSTQQ-GQNGAAAEIPKQKTFTESLALFDGRK
NCU07960.1	HPAAPSHQTSTDSTTAPKDEEHIHTATEAPASTTGAPTTTTTTPPKATFLQSLALFNGRK
FG00405.1 	KRQTPSPQRDSGEAMAQELDQHAPGFTLFPKSN-ALQPIGNSTTPKKTFLQSISLFDGRK
AN5763.1  	QHAEFRKAQDEADSREPKQSSASNGESSLDVAS-QEPPTQRSILTKVSYVQTLKLFNGRK
          	:        .   :   .  .                        : :: ::: **:***

MG05085.1 	TDESFWKLLFRPFVLLVAQPAFLW~ASLIQG1TMIGWTVFIGVILGNVFLGPPKWWNEVD
NCU07960.1	SSDSFWKLTLRPLPLLL-HPAFLW~ACLIQG~LLIGWTVFIGVLMAFFFISVPLWWDEVQ
FG00405.1 	TDERYWVLLLRPFPLLT-HPAFIW~GCLIQG~TMIGWTVFIGVIVAAIFIGPPYYWGEVD
AN5763.1  	TDEHWWKLLLRPFPLFF-HPAVLW0ACLIQG~VIIGWTVFIGVTLAAIFMSGPLWFEEDK
          	:.: :* * :**: *:  :**.:* ..****  :********* :. .*:. * :: * .

MG05085.1 	TGYAYTGPFLGALVGFAISGGLSDWSARKLTKWNNGVYEPEFRILLTIPQLVLGCAS~LY
NCU07960.1	TGYAYTGAFVGALIGFAVAGGLSDWSARLMTKWNGGVYEPEFRLVLVIPQMVLGCAG~LY
FG00405.1 	AGYTYTGPFIGAVIGFMLAGLLADTSVKYMTKLNKGIYEPEFRILLIIPMMIIGGIG~LF
AN5763.1  	TGYMYAGAFIGAMVGLVLSGLLSDFMNNIMIKFNKGRYEPEFRILLVIFQLIFCGIG1--
          	:** *:*.*:**::*: ::* *:*   . : * * * ******::* *  :::   .   

MG05085.1 	GFGATADAIYQNKADAPALLLCFGLEVAA2MVIGAVASSLYIVDAYR~DLSIEGFTCMII
NCU07960.1	GFGITLDGMLYGKYHYAVPLTFFGLEVAG~MVIGAVAGSLYIVDAYR1DIVIEGFTAMII
FG00405.1 	GFALTAPGVIEKRYPYQVPLIFFGFEVAG~MVIGAVASSLYIVDAYR1DLTIEGFTIMII
AN5763.1  	-----------------------------~-----------------~NIVIEAFTCMLI
          	                                                :: **.** *:*

MG05085.1 	FKNFFSYALTYK0AYDWWLELKTVSTPLFNIIGTVQLVICLTSIPMY1IYGKRVRSYYYR
NCU07960.1	FKNFFSFALTFK~AWPWLIQSSVKAAKIFNALGSVQLVVCLLTIPLY1IYGKRLRSFSYR
FG00405.1 	FKNFFSFMLTFF~AYNWINNGGIERTMIS--IASIQVGVCLLSIPMY1IYGKRVRAYYYR
AN5763.1  	FKNIFSFVLTFY~TYDWLVEAYVKKPFMA--ISSIQVAICALSIPMY1IFGKWNRFFFSR
          	***:**: **:  :: *  :     . :   :.::*: :*  :**:* *:**  * :  *

MG05085.1 	HDILAACGLR2VWRREGWFVVDKMKVELRPTGFKTIA
NCU07960.1	HDILELCHLR~--------------------------
FG00405.1 	HDLLAMTGLR~--------------------------
AN5763.1  	YDILKLLRLW~--------------------------
          	:*:*    *
```
